# Association Between Racial and Ethnic Diversity in Medical Specialties and Residency Application Rates

**DOI:** 10.1001/jamanetworkopen.2022.40817

**Published:** 2022-11-11

**Authors:** Max Jordan Nguemeni Tiako, Shawn Johnson, Muzzammil Muhammad, Nora Y. Osman, Sonja R. Solomon

**Affiliations:** 1 Department of Internal Medicine, Brigham and Women’s Hospital, Boston, Massachusetts; 2 Harvard Medical School, Boston, Massachusetts; 3 Yale School of Medicine, New Haven, Connecticut

## Abstract

**Importance:**

The lack of racial and ethnic diversity in the US medical profession is a well-recognized problem, and racial and ethnic representation is highly variable across the medical specialties. Residency selection is a crucial juncture at which diversity and representation in specialties can be increased.

**Objective:**

To identify factors associated with residency application rates for medical specialties by race and ethnicity.

**Design, Setting, and Participants:**

This national cross-sectional study of medical student residency applications used American Association of Medical Colleges data on 2019-2020 applicants and information about the racial and ethnic characteristics of practicing physicians (including medical school faculty) and department chairs. A total of 26 320 applicants to medical residency programs, 592 296 practicing physicians, and 2121 department chairs across the US were included. Residency application rates for 18 medical specialties were evaluated.

**Main Outcomes and Measures:**

The main outcome was the specialty representation quotient (SRQ), which estimated the extent to which students from a racial or ethnic group were overrepresented (an SRQ >1) or underrepresented (an SRQ <1) in a given specialty compared with the racial and ethnic demographic characteristics of the corresponding graduating class. Covariates included the racial and ethnic demographic characteristics of practicing physicians and department chairs by specialty based on American Association of Medical Colleges data and student academic factors (mean United States Medical Licensing Examination step 1 score, number of research experiences, and AΩA honor society membership among matched students from the previous application cycle). Multivariable logistic regression analysis was used to examine associations between these covariates and application rates by race and ethnicity.

**Results:**

Among 26 320 specialty-specific applications to medical residency programs in 18 specialties, 90 (0.3%) were from American Indian or Alaska Native students, 6718 (25.5%) were from Asian students, 2575 (9.8%) were from Black students, 1896 (7.2%) were from Hispanic students, and 15 041 (57.1%) were from White students. Among 592 296 practicing physicians, 2777 (0.5%) were American Indian or Alaska Native, 117 358 (19.8%) were Asian, 36 639 (6.2%) were Black, 41 071 (6.9%) were Hispanic, and 394 451 (66.6%) were White. Among 2121 department chairs, 5 (0.2%) were American Indian or Alaska Native, 212 (10.0%) were Asian, 86 (4.1%) were Black, 88 (4.1%) were Hispanic, and 1730 (81.6%) were White. The specialties with the greatest representation among applicants from racial and ethnic groups underrepresented in medicine (URM) were family medicine (SRQ, 1.70), physical medicine and rehabilitation (SRQ, 1.60), and obstetrics and gynecology (SRQ, 1.47). The specialties with the lowest URM representation among applicants were plastic surgery (SRQ, 0.47), otolaryngology (SRQ, 0.53), and orthopedic surgery (SRQ, 0.86). Membership in AΩA was negatively associated with SRQ among American Indian or Alaska Native students only (β = –0.11; 95% CI, –0.17 to –0.05; *P* = .002). Racial and ethnic representation among practicing physicians was positively associated with SRQ for American Indian or Alaska Native students (β = 6.05; 95% CI, 4.26-7.85; *P* < .001), Asian students (β = 0.07; 95% CI, 0.06-0.09; *P* < .001), Black students (β = 0.10; 95% CI, 0.06-0.15; *P* < .001), and URM students overall (β = 0.05; 95% CI, 0.01-0.08; *P* = .02).

**Conclusions and Relevance:**

This study’s findings suggest that the propensity of medical students, particularly those from racial and ethnic minority groups, to apply to a given specialty for residency was associated with the representation of their racial or ethnic group among the specialty’s practicing physicians. Future work to characterize the mechanisms of occupational sorting may guide interventions to improve equity within the physician workforce.

## Key Points

**Question:**

Is there an association between racial and ethnic representation in medical specialties and residency application rates by racial and ethnic group?

**Findings:**

In this cross-sectional study including 26 416 applicants to medical residency programs, 592 296 practicing physicians (including medical school faculty), and 2121 department chairs in the US, racial and ethnic representation among practicing physicians was positively associated with residency application rates for medical specialties by racial and ethnic group.

**Meaning:**

These findings suggest that racial and ethnic representation in medical specialties may have implications for minority medical students’ pursuit of those specialties.

## Teaser

This cross-sectional study uses data from the American Association of Medical Colleges to identify representational factors associated with residency application rates for medical specialties by race and ethnicity.

## Introduction

According to the Liaison Committee on Medical Education standards on diversity, US medical schools have a mandate to “have policies and practices to achieve appropriate diversity among its students, faculty, staff, and other members of its academic community, and must engage in ongoing, systematic, and focused efforts to attract and retain students, faculty, staff, and others from demographically diverse backgrounds.”^1^ Despite a US population that is projected to be majority-minority (defined as comprising <50% non-Hispanic White individuals) by 2030, there remains a lack of racial and ethnic diversity and representation among US medical trainees, practicing physicians, and leaders of academic medical programs.^2,3,4^ There is substantial variation in racial and ethnic representation across specialties; primary care specialties and obstetrics and gynecology are the most racially and ethnically diverse specialties, whereas ophthalmology, radiation oncology, and orthopedic surgery are among the least diverse.^5,6^ Racial and ethnic diversity at the specialty level is essential because it may shape access to health care among underserved communities; racial and ethnic minority medical students have reported greater intent to practice in underserved settings, and there is a skew in their representation across specialties, favoring primary care specialties. In addition, among those interested in surgical specialties, racial and ethnic minority medical students are more likely to report an intent to practice in underserved areas.^7^

When asked in 2020, most medical students reported that fit with personality, interest, and skill strongly influenced their specialty choice.^8^ More than one-half of respondents cited role model influences as a vital factor in this choice.^8^ Other factors, such as specialty competitiveness and extent of exposure in medical school, are known to play a role in students’ specialty choice.^9^ Factors including fit with personality and role model influence are likely subject to individual and structural biases.^10^ Students from racial and ethnic groups who are underrepresented in medicine (URM) have reported that a lack of faculty diversity contributes to a lack of mentorship.^11,12^ Studies have explored variations in minority students’ specialty choices and associated factors.^13,14^ A recent study found that sex representation within a given specialty was associated with greater interest in the specialty among female students.^15^ Similarly, another study found that medical schools with a greater number of URM faculty in orthopedic surgery produced relatively more URM applicants in orthopedic surgery.^16^ However, the extent to which racial and ethnic representation within a given specialty may be associated with medical students’ specialty choices across specialties remains unclear. In this study, we built on previous work describing occupational sorting (a systemic pattern of distribution of workers across and within occupations according to demographic characteristics, often as a consequence of active processes such as discrimination, segregation, and resource allocation among minority physicians) to (1) examine the association between racial and ethnic representation among practicing physicians (including medical school faculty) and leadership (department chairs) at medical schools by specialty and (2) assess residency application rates by student race and ethnicity at the national level.

## Methods

### Study Type

This cross-sectional study used publicly available national data from medical students’ residency applications and information on the characteristics of practicing physicians (including medical school faculty) and department chairs by racial and ethnic group. The study followed the Strengthening the Reporting of Observational Studies in Epidemiology (STROBE) reporting guideline for cross-sectional studies.^17^ This study was approved by the institutional review board of Yale University with a waiver of informed consent due to the use of publicly available data.

### Data

We used the 2019 Facts: Electronic Residency Application Service database of the American Association of Medical Colleges (AAMC) to obtain information on residency applicants from US schools granting doctor of medicine degrees by race and ethnicity for the 2019-2020 academic year.^18^ Indices reflecting specialty-specific competitiveness (matched applicants’ mean step 1 and step 2 scores on the United States Medical Licensing Examination [USMLE] from the previous year and number of research experiences) were obtained from the AAMC 2019 Report on Residents.^19^ These data reflected the previous application season’s cohort, which likely was a factor in applicants’ specialty choices based on perceived competitiveness. The specialty’s inclusion in core clinical clerkships at most medical schools per AAMC curriculum reports reflected exposure to a specialty.

We obtained physician practice specialty and racial and ethnic demographic characteristics from the AAMC 2018 US physician workforce data in the Diversity in Medicine: Facts and Figures 2019 report.^20^ We used department chairs by specialty and by race and ethnicity to reflect specialty leadership, as reported in the 2019 AAMC Faculty Roster of US Medical Schools.^21^

We excluded specialties, such as ophthalmology and urology, that do not participate in the Electronic Residency Application Service. For simplicity in our model, we also excluded applications to combined-specialty residency programs, such as internal medicine-pediatrics and emergency medicine-internal medicine. Surgical residencies with categorical tracks, such as vascular surgery, plastic surgery, and neurosurgery, were included in the surgery category based on data availability. After exclusions, a total of 18 specialties were evaluated: anesthesiology, dermatology, emergency medicine, family medicine, internal medicine, neurological surgery, neurology, obstetrics and gynecology, orthopedic surgery, otolaryngology, pathology–anatomic and clinical, pediatrics, plastic surgery–integrated, psychiatry, physical medicine and rehabilitation, radiology, surgery–general, and vascular surgery–integrated.

### Outcome and Covariates

The primary outcome was the specialty representation quotient (SRQ), which estimated the extent to which students from a racial or ethnic group were over- or underrepresented in a given specialty compared with the racial and ethnic demographic characteristics of the corresponding graduating class. The SRQ is an adaptation of a quotient previously used to characterize racial and ethnic minority representation in medical schools relative to the general population.^2^ The SRQ was calculated as the percentage of applicants from a racial or ethnic group to a given specialty divided by the percentage of graduating medical students from the same racial or ethnic group. An SRQ greater than 1 suggested overrepresentation, whereas an SRQ less than 1 suggested underrepresentation. We also used the variance in SRQs across specialties by racial or ethnic group to assess the evenness of the distribution of interest across specialties. A variance approaching 0 suggested a more even distribution across specialties. We also evaluated the SRQ in composite for URM students.

Race and ethnicity data are defined and reported in the AAMC database based on self-reporting of students and practicing physicians.^20^ The AAMC defines URM populations as racial and ethnic groups that are underrepresented in the medical profession relative to their numbers in the general population. For the purpose of this study, we defined URM populations as American Indian or Alaska Native, Black, and Hispanic students, practicing physicians, and department chairs.

Covariates were selected based on previous evidence of association with applications and successful matches. They were limited to the leading publicly available quantifiable factors that program directors cited as important when rating applicants.^22^ These factors included specialty-specific mean USMLE step 1 scores, membership in AΩA (medical honor society), mean number of research experiences (as reported in the AAMC report on residents), whether the specialty was part of the core required clerkships at most medical schools based on AAMC data, number of positions offered, racial and ethnic representation among practicing physicians by specialty, and racial and ethnic representation among medical school department chairs and practicing physicians by specialty. Data on age and sex were not collected; although the AAMC provides these individual-level data for practicing physicians and department chairs, the same data were not available for residency applicants at the individual level. Many surgical specialty programs we studied did not have department chair data; we estimated that it was appropriate to assign surgery department chairs to surgical specialties (vs general surgery) because at academic medical centers, chiefs of surgery and surgeons-in-chief generally oversee all surgical specialties. Step 2 scores from the USMLE were not included in our model because it is common for medical students to apply for residency before they complete this examination.

### Statistical Analysis

Specialty-specific data were first analyzed descriptively. We performed a multivariable linear regression analysis to assess the association between preselected covariates and the SRQ for each specialty. We used a linear regression model for this outcome because our dependent variable (SRQ) was continuous. Results from the regression analysis were reported using β coefficients, which represent the extent of change in the outcome variable for every 1-unit change in the independent variable. All statistical tests were 2-tailed, and statistical significance was defined as *P* < .05. All analyses were performed using Stata software, version 16 (StataCorp LLC).

## Results

Among 26 320 specialty-specific applications to medical residency programs in 18 specialties, 90 (0.3%) were from American Indian or Alaska Native students, 6718 (25.5%) were from Asian students, 2575 (9.8%) were from Black students, 1896 (7.2%) were from Hispanic students, and 15 041 (57.1%) were from White students. Among 592 296 practicing physicians, 2777 (0.5%) were American Indian or Alaska Native, 117 358 (19.8%) were Asian, 36 639 (6.2%) were Black, 41 071 (6.9%) were Hispanic, and 394 451 (66.6%) were White. Among 2121 department chairs, 5 (0.2%) were American Indian or Alaska Native, 212 (10.0%) were Asian, 86 (4.1%) were Black, 88 (4.1%) were Hispanic, and 1730 (81.6%) were White. Additional racial and ethnic demographic characteristics of department chairs, practicing physicians, and applicants are shown in eTables 1 to 3 in the Supplement, and information about applicants by specialty is shown in eTable 4 in the Supplement.

All included specialties with respective SRQs by racial and ethnic group are shown in Table 1 and the Figure. Of the 18 medical specialties evaluated, those with the greatest URM representation among applicants were family medicine (SRQ, 1.70), physical medicine and rehabilitation (SRQ, 1.60), and obstetrics and gynecology (SRQ, 1.47). The specialties with the lowest URM representation among applicants were plastic surgery (SRQ, 0.47), otolaryngology (SRQ, 0.53), and orthopedic surgery (SRQ, 0.86). The variance in SRQ was 1.27 among American Indian or Alaska Native applicants, 0.05 among Asian applicants, 0.19 among Black applicants, 0.06 among Hispanic applicants, and 0.01 among White applicants.

**Table 1.** Specialty Representation Quotient by Specialty and Applicant Race and Ethnicity

Specialty	SRQ by applicant race and ethnicity^a^					
	American Indian or Alaska Native	Asian	Black	Hispanic	White	URM
Anesthesiology	1.22	1.07	1.55	1.00	0.90	1.26
Dermatology	1.14	0.99	0.95	1.17	0.94	1.03
Emergency medicine	1.65	0.66	1.07	1.19	1.10	1.10
Family medicine	2.83	0.76	1.96	1.45	0.92	1.70
Internal medicine	1.05	1.27	1.08	1.07	0.87	1.05
Neurological surgery	1.46	1.03	1.12	1.04	0.90	1.06
Neurology	0	1.26	1.05	1.33	0.82	1.13
Obstetrics and gynecology	1.23	0.69	1.83	1.14	1.00	1.47
Orthopedic surgery	0.41	0.69	1.02	0.74	1.16	0.86
Otolaryngology	0	1.11	0.47	0.65	1.04	0.53
Pathology–anatomic and clinical	0	0.78	1.32	0.83	1.06	1.05
Pediatrics	1.14	0.83	1.24	1.18	1.04	1.18
Plastic surgery–integrated	0	1.02	0.42	0.58	1.09	0.47
Psychiatry	1.68	0.93	1.51	1.23	0.90	1.36
Physical medicine and rehabilitation	2.46	1.43	1.84	1.36	0.91	1.60
Radiology	1.40	1.22	0.99	1.01	0.89	0.98
Surgery–general	1.44	0.95	1.34	1.16	0.94	1.23
Vascular surgery–integrated	4.43	1.01	1.70	1.41	0.75	1.58

Abbreviations: SRQ, specialty representation quotient; URM, underrepresented in medicine.

^a^ SRQ is an estimate of the extent to which students from a racial or ethnic group were over- or underrepresented in a given specialty in comparison with the racial and ethnic demographic characteristics of the corresponding graduating class. The SRQ was calculated as the percentage of applicants from a racial or ethnic group to a given specialty divided by the percentage of graduating medical students from the same racial or ethnic group. An SRQ greater than 1 suggests overrepresentation, whereas an SRQ less than 1 suggests underrepresentation.

**Figure.**
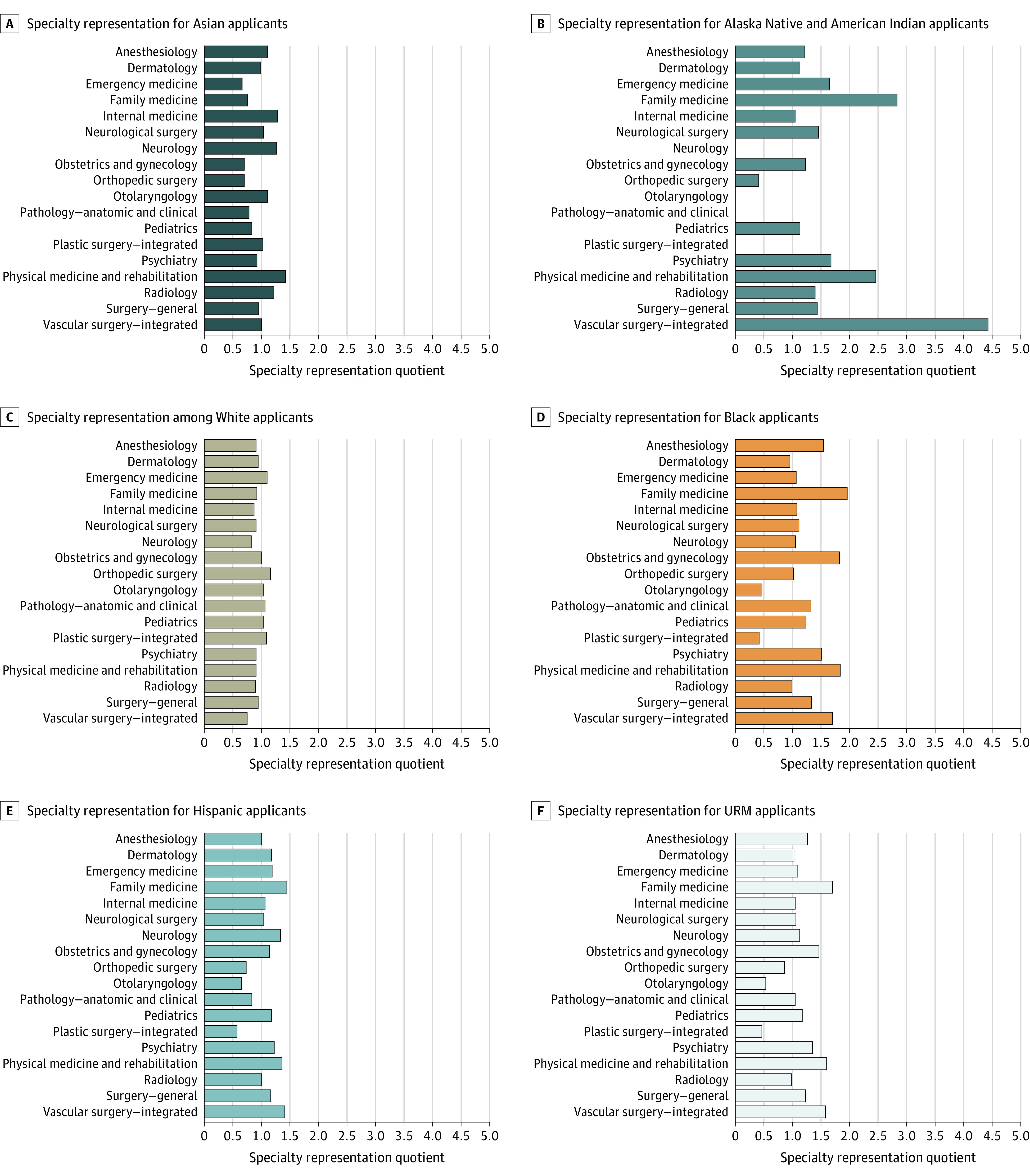
Racial Representation Across Specialties by Racial and Ethnic Group Based on Specialty Representation Quotient

In our multivariable linear regression analysis (Table 2), AΩA membership among matched applicants from the previous year was negatively associated with SRQ among American Indian or Alaska Native students only (β = –0.11; 95% CI, –0.17 to –0.05; *P* = .002). In other words, each percentage point increase in the number of matched applicants who were AΩA members was associated with a decrease in SRQ of 0.11 among American Indian or Alaska Native students. Racial and ethnic representation among practicing physicians was positively associated with SRQ for American Indian or Alaska Native students (β = 6.05; 95% CI, 4.26-7.85; *P* < .001), Asian students (β = 0.07; 95% CI, 0.06-0.09; *P* < .001), Black students (β = 0.10; 95% CI, 0.06-0.15; *P* < .001), and URM students overall (β = 0.05; 95% CI, 0.01-0.08; *P* = .02). In other words, each percentage point increase in the number of American Indian or Alaska Native, Asian, Black, and URM practicing physicians was associated with an increase in SRQ of 6.05 among American Indian or Alaska Native students, 0.07 among Asian students, 0.10 among Black students, and 0.05 among URM students overall. No association with SRQ was found among White applicants (β = 0.01; 95% CI, –0.01 to 0.02; *P* = .27).

**Table 2.** Linear Regression Analysis of the Association Between Key Covariates and Specialty Representation Quotient by Applicant Race and Ethnicity

Covariate	Association with SRQ by applicant race and ethnicity											
	American Indian or Alaska Native		Asian		Black		Hispanic		White		URM	
	β (95% CI)	*P* value	β (95% CI)	*P* value	β (95% CI)	*P* value	β (95% CI)	*P* value	β (95% CI)	*P* value	β (95% CI)	*P* value
USMLE step 1 score	–0.04 (–0.10 to 0.02)	.21	0.01 (0 to 0.01)	.22	–0.03 (–0.06 to 0)	.08	–0.03 (–0.06 to 0)	.03	0 (–0.01 to 0.02)	.75	–0.02 (–0.05 to 0.01)	.10
AΩA membership	–0.11 (–0.17 to –0.05)	.002	0 (–0.01 to 0.01)	.99	–0.01 (–0.04 to 0.01)	.34	–0.01 (–0.03 to 0.01)	.30	0.01 (0.01 to 0.02)	.003	–0.01 (–0.04 to 0.01)	.35
Core rotation status	–0.40 (–1.00 to 0.20)	.17	0.09 (–0.03 to 0.22)	.13	–0.29 (–0.54 to –0.05)	.02	0.25 (–0.02 to 0.53)	.07	–0.03 (–0.17 to 0.11)	.64	–0.09 (–0.31 to 0.14)	.41
Research experiences	1.69 (1.00 to 2.38)	<.001	0.05 (–0.01 to 0.21)	.46	0.11 (–0.12 to 0.35)	.32	0.37 (0.10 to 0.64)	.01	–0.19 (–0.27 to –0.11)	<.001	0.16 (–0.11 to 0.44)	.21
Practicing physicians (corresponding race and ethnicity)	6.05 (4.26 to 7.85)	<.001	0.07 (0.06 to 0.09)	<.001	0.10 (0.06 to 0.15)	<.001	0.08 (–0.05 to 0.22)	.20	0.01 (–0.01 to 0.02)	.27	0.05 (0.01 to 0.08)	.02
Department chairs (corresponding race and ethnicity)	0.09 (0.01 to 0.18)	.03	–0.02 (–0.04 to 0)	.10	0.02 (–0.02 to 0.06)	.22	–0.10 (–0.18 to –0.01)	.03	0 (–0.02 to 0.01)	.49	0 (–0.03 to 0.03)	.84
*R* ^2^	0.91	NA	0.93	NA	0.85	NA	0.69	NA	0.72	NA	0.78	NA

Abbreviations: NA, not applicable; SRQ, specialty representation quotient; URM, underrepresented in medicine; USMLE, United States Medical Licensing Examination.

Department chair racial and ethnic representation was positively associated with SRQ among American Indian or Alaska Native applicants (β = 0.09; 95% CI, 0.01-0.18; *P* = .03) and negatively associated with SRQ among Hispanic applicants (β = –0.10; 95% CI, –0.18 to –0.01; *P* = .03). No association with SRQ was found among Asian applicants (β = –0.02; 95% CI, –0.04 to 0; *P* = .10), Black applicants (β = 0.02; 95% CI, –0.02 to 0.06; *P* = .22), White applicants (β = 0; 95% CI, –0.02 to 0.01; *P* = .49), or URM applicants (β = 0; 95% CI, –0.03 to 0.03; *P* = .84).

## Discussion

This cross-sectional study had 2 main findings. First, in most cases, representation of a given racial or ethnic group among practicing physicians in any specialty was positively associated with residency application rates among students of the same racial or ethnic group, including American Indian or Alaska Native, Asian, and Black applicants. Second, for SRQ variance, racial and ethnic representation across specialties was most evenly distributed among Asian and White applicants and least evenly distributed among American Indian or Alaska Native and Black applicants.

Our findings regarding the associations of racial and ethnic representation among practicing physicians with student application rates were consistent with previous studies on the role of faculty demographic characteristics in specialty interest among students.^15,16^ The specialties with greater SRQs among URM students, such as obstetrics and gynecology and family medicine, have previously been identified as the most racially diverse specialties at the graduate medical education level.^5^ Application rates may also reflect a specialty’s commitment to recruiting a diverse workforce as a function of the issues encountered by their patient populations.

Many factors may explain our findings. First, a students’ career trajectory is shaped by many aspects of medical education, including mentorship, experiences of discrimination, and positive and negative role modeling in the learning environment.^23,24^ Medical students from URM populations, in particular, are more likely to lack mentorship and to report reliance on URM practicing physicians for support and career guidance.^11,12,13,25^ Specialties with more diversity may offer more mentorship to URM students, thus influencing their career choices. The learning environment also has implications for professional development and career choice. Students from URM groups have reported experiencing discrimination at greater rates,^13,26^ and studies have found racial disparities in clerkship grades^27,28,29^ and medical student evaluations.^30^ Stereotype threat (ie, fear of confirming a primarily negative perception about one’s racial, ethnic, or other social group), which is prevalent among Asian and Black students,^31^ is more likely to occur in less diverse settings, with consequences for performance, grading, and effective educational alliances.^32^ This theory is supported by research focusing on medical school type as a proxy for racial climate; a recent study^33^ found that Black medical students at historically Black medical schools reported a greater sense of belonging and confidence in their scholastic abilities and were less likely to report changes in their specialty intentions over time compared with their Black counterparts at predominantly White medical schools, which is likely associated with differences in the racial environment.

Factors external to medical schools may, however, have positive implications for URM students’ interest in a given specialty. Examples include mentorship sought through professional organizations designed for racial and ethnic or sex and gender minority individuals and specialty-specific programming to support URM trainees. For example, Nth Dimensions,^34^ a nonprofit organization aiming to increase the diversity of female and URM individuals in specialty medical fields, offers minority medical students early exposure to surgical specialties during the first summer of medical school through an internship and structured mentorship program. These efforts rely on the ability and willingness of practicing physicians to mentor medical students. Underrepresented practicing physicians have described the cost of a minority tax (ie, the burden of extra duties and responsibilities minority practicing physicians are asked to perform to increase institutional diversity),^35^ in part related to the high demands on their time.

Our findings have implications for the physician workforce and warrant further identification of the mechanisms associated with occupational sorting along the educational continuum. The finding that racial and ethnic representation across specialties was most unevenly distributed among American Indian or Alaska Native and Black applicants suggests a complex racialized occupational sorting process that reinforces societal inequities within the physician workforce.^36^ The variable sorting of Black students, who are already overrepresented among those economically disadvantaged,^37,38^ into medical specialties that are less lucrative may perpetuate economic and racial inequities within the physician workforce, further amplified by racial disparities in earnings within specialties.^39,40,41,42^ Beyond the inequities within the physician workforce, these disparities in representation may have second-order consequences for patient access to subspecialty care, given that URM medical students and physicians are more likely to report an interest in serving underserved communities.^43^ It is notable that Black medical students accounted for approximately 9.8% of all applications but made up fewer than 7% of all graduating medical students in the corresponding year. This suggests that Black medical students apply to multiple specialties, either out of greater uncertainty or concerns about successfully matriculating in their preferred specialty. Indeed, studies have reported a more substantial application-matriculation gap among URM trainees applying to surgical specialties than among Asian and White trainees.^3,44,45^

### Strengths and Limitations

This study has strengths and limitations. We used national cross-sectional data from a single application cycle because of availability. However, studies have found that patterns in racial representation among medical school enrollees and practicing physicians are slow to change over a few years.^2,6^ Because these data are observational and not longitudinal, we cannot make causal inferences. Although the specialties included in our study account for more than 80% of all applications to residency programs, our analysis was limited to specialties for which data on practicing physicians and department chairs were available by racial and ethnic group. Our findings specific to URM applicants may be conservative estimates because the specialties excluded, including radiation oncology, ophthalmology, and urology,^46,47,48^ are among the least diverse. We use application rather than matriculation data, recognizing that students may apply to multiple specialties out of interest or necessity and may ultimately matriculate into only 1 specialty. Our model may have excluded unknown confounders; however, given our use of national data, we made sure to include variables known to be associated with student choices based on competitiveness. In addition, we did not account for racial and ethnic representation among residents because of collinearity between medical student and resident racial and ethnic demographic characteristics at the national scale.^49^

## Conclusions

In this cross-sectional study, racial and ethnic representation among practicing physicians was positively associated with residency application rates at the national level, especially among racial and ethnic minority students. As medical schools and residency program leadership commit to addressing the implications of structural racism for trainees’ career advancement, they should also attend to and rectify these important associations. Future studies should further investigate the mechanisms behind these observations, including qualitative approaches, and evaluate structural interventions that may address the consequences of racism for medical students’ career trajectories to improve equity within the physician workforce.

## Notes

Supplement. **eTable 1.** Department Chairs by Specialty and by Race and Ethnicity **eTable 2.** Practicing Physicians by Specialty and by Race and Ethnicity **eTable 3.** Residency Applicants by Specialty and by Race and Ethnicity **eTable 4.** Specialty-Specific Academic Information
