# Supplementary material for: Association Between Racial and Ethnic Diversity in Medical Specialties and Residency Application Rates
Source: JAMA Netw Open. 2022 Nov 11;5(11):e2240817. doi: 10.1001/jamanetworkopen.2022.40817 (PMC9652751; doi:10.1001/jamanetworkopen.2022.40817)
Supplement: Supplement. — eTable 1. Department Chairs by Specialty and by Race and Ethnicity eTable 2. Practicing Physicians by Specialty and by Race and Ethnicity eTable 3. Residency Applicants by Specialty and by Race and Ethnicity eTable 4. Specialty-Specific Academic Information [file jamanetwopen-e2240817-s001.pdf]

## Supplemental Online Content

Nguemeni Tiako MJ, Johnson S, Muhammad M, Osman NY, Solomon SR. Association between racial and ethnic diversity in medical specialties and residency application rates. *JAMA Netw Open*. 2022;5(11):e2240817. doi:10.1001/jamanetworkopen.2022.40817

**eTable 1.** Department Chairs by Specialty and by Race and Ethnicity

**eTable 2.** Practicing Physicians by Specialty and by Race and Ethnicity

**eTable 3.** Residency Applicants by Specialty and by Race and Ethnicity

**eTable 4.** Specialty-Specific Academic Information

This supplemental material has been provided by the authors to give readers additional information about their work.

© 2022 Nguemeni Tiako MJ et al. *JAMA Network Open*.

**eTable 1.** Department Chairs by Specialty and by Race and Ethnicity

| Chairs | Black | Hispanic | American Indian/Alaska Native (AIAN) | Asian | White |
|---|---|---|---|---|---|
| Anesthesiology | 2 (1.7%) | 2 (1.7%) | 0 (0%) | 5 (4.2%) | 105 (87.5%) |
| Dermatology | 2 (2.4%) | 4 (4.8%) | 0 (0%) | 10 (12.0%) | 66 (79.5%) |
| Emergency Medicine | 4 (3.5%) | 3 (2.6%) | 1 (7.7%) | 6 (5.3%) | 99 (86.8%) |
| Family Medicine | 16 (11.7%) | 6 (4.4%) | 1 (2.4%) | 6 (4.4%) | 105 (76.6%) |
| Internal Medicine | 9 (5.0%) | 9 (5.0%) | 0 (0%) | 20 (11.2%) | 136 (76.0%) |
| Neurology | 1 (0.8%) | 5 (3.9%) | 0 (0%) | 13 (10.2%) | 101 (78.9%) |
| Obstetrics and Gynecology | 10 (6.4%) | 8 (5.1%) | 0 (0%) | 10 (6.4%) | 123 (78.8%) |
| Orthopedic Surgery | 4 (3.3%) | 2 (1.7%) | 0 (0%) | 5 (4.2%) | 107 (89.2%) |
| Otolaryngology | 1 (1.2%) | 3 (3.4%) | 0 (0%) | 12 (13.8%) | 70 (80.5%) |
| Pathology-Anatomic and Clinical | 1 (1.0%) | 4 (4.1%) | 0 (0%) | 13 (13.4%) | 79 (81.4%) |
| Pediatrics | 7 (4.5%) | 8 (5.2%) | 2 (4.8%) | 11 (7.1%) | 119 (77.3%) |
| Physical Medicine and Rehabilitation | 2 (3.3%) | 2 (3.3%) | 0 (0%) | 7 (11.5%) | 46 (75.4%) |
| Psychiatry | 4 (2.6%) | 9 (5.8%) | 1 (2.8%) | 12 (7.7%) | 121 (78.1%) |
| Radiology | 9 (4.2%) | 5 (2.3%) | 0 (0%) | 34 (15.9%) | 160 (74.8%) |
| Surgery | 14 (3.7%) | 18 (4.7%) | 0 (0%) | 48 (12.6%) | 293 (76.7%) |

© 2022 Nguemeni Tiako MJ et al. *JAMA Network Open*.

**eTable 2.** Faculty by Specialty and by Race and Ethnicity

| Faculty | **Black** | **Hispanic** | American Indian/Alaska Native (AIAN) | **Asian** | **White** |
|---|---|---|---|---|---|
| Anesthesiology | 1955 (4.7%) | 2089 (5.0%) | 170 (0.4%) | 6555 (15.6%) | 26059 (62.1%) |
| Dermatology | 422 (3.4%) | 537 (4.4%) | 39 (0.3%) | 1519 (12.4%) | 8108 (66.0%) |
| Emergency Medicine | 1975 (4.5%) | 2309 (5.3%) | 207 (0.5%) | 4277 (9.8%) | 30311 (69.3%) |
| Family Medicine | 6581 (5.7%) | 7647 (6.5%) | 786 (0.7%) | 15323 (13.2%) | 66898 (57.5%) |
| Internal Medicine | 11965 (5.1%) | 13637 (5.8%) | 635 (0.3%) | 55763 (23.8%) | 109946 (46.9%) |
| Neurological Surgery | 216 (3.8%) | 282 (5.0%) | 17 (0.3%) | 820 (14.5%) | 3618 (64.1%) |
| Neurology | 349 (2.5%) | 769 (5.5%) | 23 (0.2%) | 2371 (17.0%) | 7942 (57.1%) |
| Obstetrics and Gynecology | 4055 (9.6%) | 2849 (6.7%) | 187 (0.4%) | 4414 (10.4%) | 25215 (59.7%) |
| Orthopedic Surgery | 513 (2.7%) | 523 (2.8%) | 74 (0.4%) | 1255 (6.6%) | 13443 (70.7%) |
| Otolaryngology | 230 (2.4%) | 339 (3.5%) | 20 (0.2%) | 1328 (13.8%) | 6408 (66.5%) |
| Pediatrics | 3731 (6.2%) | 4281 (7.2%) | 219 (0.4%) | 8268 (13.8%) | 32735 (54.7%) |
| Physical Medicine and Rehabilitation | 612 (6.4%) | 644 (6.7%) | 41 (0.4%) | 1975 (20.5%) | 5312 (55.1%) |
| Plastic Surgery-Integrated | 206 (2.9%) | 385 (5.3%) | 26 (0.4%) | 890 (12.3%) | 4606 (63.8%) |
| Psychiatry | 1813 (4.7%) | 2192 (5.7%) | 139 (0.4%) | 5146 (13.4%) | 20544 (53.3%) |
| Radiology | 658 (2.4%) | 1106 (4.0%) | 75 (0.3%) | 4240 (15.2%) | 18252 (65.5%) |
| Surgery-General | 1358 (5.4%) | 1482 (5.9%) | 119 (0.5%) | 3214 (12.7%) | 15054 (59.5%) |

© 2022 Nguemeni Tiako MJ et al. *JAMA Network Open*.

**eTable 3.** Residency Applicants by Specialty and by Race and Ethnicity

Commented [MJNT1]: Thoracic surgery was excluded from the analysis due to missing data, so deleting from this table

| Applicants | Black | Hispanic | American Indian/Alaska Native (AIAN) | Asian | White |
|---|---|---|---|---|---|
| Anesthesiology | 191 (10.2%) | 103 (5.5%) | 5 (0.3%) | 442 (23.7%) | 885 (47.5%) |
| Dermatology | 50 (6.3%) | 52 (6.5%) | 2 (0.3%) | 175 (21.9%) | 398 (49.9%) |
| Emergency Medicine | 155 (7.0%) | 145 (6.6%) | 8 (0.4%) | 324 (14.7%) | 1282 (58.2%) |
| Family Medicine | 395 (13.0%) | 245 (8.0%) | 19 (0.6%) | 515 (16.9%) | 1481 (48.6%) |
| Internal Medicine | 614 (7.1%) | 513 (5.9%) | 20 (0.2%) | 2421 (28.0%) | 3978 (46.1%) |
| Neurological Surgery | 23 (7.4%) | 18 (5.8%) | 1 (0.3%) | 71 (22.8%) | 148 (47.4%) |
| Neurology | 51 (7.0%) | 54 (7.4%) | 0 (0%) | 204 (27.8%) | 320 (43.6%) |
| Obstetrics and Gynecology | 178 (12.1%) | 93 (6.3%) | 4 (0.3%) | 225 (15.3%) | 778 (52.8%) |
| Orthopedic Surgery | 74 (6.7%) | 45 (4.1%) | 1 (0.1%) | 169 (15.3%) | 674 (61.2%) |
| Otolaryngology | 13 (3.1%) | 15 (3.6%) | 0 (0%) | 102 (24.5%) | 228 (54.8%) |
| Pathology-Anatomic and Clinical | 34 (8.7%) | 18 (4.6%) | 0 (0%) | 67 (17.2%) | 219 (56.2%) |
| Pediatrics | 196 (8.2%) | 157 (6.6%) | 6 (0.3%) | 437 (18.3%) | 1312 (54.9%) |
| Plastic Surgery-Integrated | 6 (2.8%) | 7 (3.2%) | 0 (0%) | 49 (22.7%) | 125 (57.9%) |
| Psychiatry | 162 (10.0%) | 111 (6.8%) | 6 (0.40%) | 334 (20.6%) | 768 (47.4%) |
| Physical Medicine and Rehabilitation | 45 (12.2%) | 28 (7.6%) | 2 (0.5%) | 117 (31.6%) | 178 (48.1%) |
| Radiology | 85 (6.5%) | 73 (5.6%) | 4 (0.3%) | 352 (27.1%) | 614 (47.2%) |
| Surgery-General | 280 (8.8%) | 203 (6.4%) | 10 (0.3%) | 668 (21.1%) | 1572 (49.7%) |
| Vascular Surgery-Integrated | 23 (11.2%) | 16 (7.8%) | 2 (1.0%) | 46 (22.4%) | 81 (39.5%) |
| Total | 2575 (9.8%) | 1894 (7.25%) | 90 (0.3%) | 6718 (25.5%) | 15041 (57.1%) |

© 2022 Nguemeni Tiako MJ et al. *JAMA Network Open*.

**eTable 4.** Specialty-Specific Academic Information

| Specialty | Positions Offered | Average Step1 score | Average Step2CK score | Average Number of Research experiences | Percent matriculants that are AOA members |
|---|---|---|---|---|---|
| Anesthesiology | 1,337 | 230.6 | 241.3 | 2.4 | 10.5 |
| Emergency Medicine | 2,488 | 231.1 | 244.2 | 2 | 12.4 |
| Family Medicine | 4,107 | 215.9 | 231.1 | 1.6 | 7.4 |
| Internal Medicine | 8562 | 230.4 | 240.5 | 2.1 | 16.7 |
| Neurological Surgery | 232 | 243.7 | 247.8 | 4.8 | 31.9 |
| Neurology | 617 | 230 | 239.6 | 2.8 | 14.4 |
| Obstetrics and Gynecology | 1,395 | 228.4 | 244.7 | 2.9 | 16.2 |
| Orthopedic Surgery | 755 | 246.2 | 253.4 | 4 | 40.4 |
| Otolaryngology | 328 | 246.7 | 252.1 | 4.9 | 40.2 |
| Pathology-Anatomic and Clinical | 601 | 227.5 | 234.5 | 2.3 | 8.8 |
| Pediatrics | 2,847 | 226 | 240 | 2.3 | 14.6 |
| Physical Medicine and Rehabilitation | 138 | 224.9 | 236.5 | 2.4 | 5.3 |
| Plastic Surgery-Integrated | 172 | 246.5 | 252.5 | 4.7 | 44.5 |
| Psychiatry | 3.5 | 221.4 | 234.3 | 2.3 | 6.8 |
| Surgery-General | 1,432 | 235.1 | 246.9 | 3 | 18.7 |
| Vascular Surgery-Integrated | 66 | 235 | 242.1 | 4.4 | 17.4 |
| Dermatology | 447 | 249 | 256 | 5.2 | 49.1 |
| Radiology | 965 | 240 | 249 | 3.7 | 15.8 |

© 2022 Nguemeni Tiako MJ et al. *JAMA Network Open*.
